# A comparative analysis of core needle biopsy and repeat fine needle aspiration in patients with inconclusive initial cytology of thyroid nodules

**DOI:** 10.3389/fendo.2024.1309005

**Published:** 2024-01-30

**Authors:** Xuejiao Su, Can Yue, Wanting Yang, Buyun Ma

**Affiliations:** Department of Medical Ultrasound, West China Hospital of Sichuan University, Chengdu, Sichuan, China

**Keywords:** thyroid nodules, inconclusive initial cytological diagnosis, core needle biopsy, repeat fine-needle aspiration, ultrasound

## Abstract

**Purpose:**

To assess and compare the effectiveness of ultrasound-guided core needle biopsy (CNB) in comparison to repeat fine-needle aspiration(rFNA) for thyroid nodules that yield inconclusive results following the initial fine-needle aspiration (FNA).

**Methods:**

A cohort of 471 patients who received an inconclusive cytological diagnosis following the initial FNA were included in this study. These patients subsequently underwent either CNB (n=242) or rFNA (n=229). The inconclusive FNA results encompassed categories I, III, and IV of The Bethesda System for Reporting Thyroid Cytopathology(TBSRTC), as well as the ultrasound images indicating malignancy despite FNA results falling under TBSRTC category II. This study assessed the sampling satisfaction rate, diagnostic efficacy, and complications associated with CNB compared to rFNA. Additionally, the impact of repeat puncture time and nodule size on diagnostic efficacy was analyzed.

**Results:**

Following repeat punctures, the satisfaction rate of the CNB sampling was found to be significantly higher than that of rFNA (83.9% vs 66.8%). The diagnostic rate in the CNB group was significantly greater compared to that of the rFNA group (70.7% vs 35.8%). In patients with nodule maximum diameters ranging from 5 mm to 20 mm, the diagnostic accuracy was significantly higher in the CNB group compared to that in the rFNA group. In patients with intervals less than 90 days, between 90 days and one year, the diagnostic rate in the CNB group was found to be higher compared to that in the rFNA group. In CNB, not immediately adjacent to the capsule was a risk factor for nodular puncture bleeding (37.0% vs 22.7%.)

**Conclusion:**

CNB demonstrated higher rates of satisfaction and diagnosis compared to the rFNA. The diagnostic effectiveness of CNB was not influenced by the time interval or the size of the thyroid nodule. Therefore, in cases where the initial FNA diagnosis of thyroid nodules is inconclusive, CNB should be considered as a viable option for re-puncture.

## Introduction

Thyroid nodules are prevalent clinical conditions, affecting approximately 10% to 70% of individuals, found particularly in women and the elderly (1). FNA cytology is a commonly employed and minimally invasive technique for assessing thyroid lesions (1, 2), offering high diagnostic accuracy and safety. However, inconclusive results still occur (3), including TBSRTC category I, III, or IV, as well as the ultrasound images indicating malignancy despite FNA results falling under category II. The American Thyroid Association (ATA) guideline recommends rFNA in such cases, yet rFNA yields nondiagnostic results in 1% to 7% of all cases and indeterminate results in 3.8% to 31.0% of the cases (4, 5). Given the variable risk of malignancy associated with these inconclusive thyroid nodules, other guidelines (6) recommend the evaluation of these nodules by diagnostic surgery, but the majority (70% to 80%) of thyroid nodules are benign based on surgical histology (7), while surgery causes greater trauma and economic stress.

Therefore, it has been proposed that CNB can be used as another safe and effective diagnostic tool for the diagnosis of thyroid nodules with inconclusive cytological diagnosis to prevent unnecessary surgery (8, 9). Several studies have reported that CNB was performed after the initial FNA revealed non-diagnostic results, and the uncertainty of secondary diagnostic biopsy results for thyroid nodules was significantly reduced (5, 10). The Korean Thyroid Society suggests that considering CNB as a viable option for thyroid nodules either lacks a definitive diagnosis or yields indeterminate results from previous cytology (10, 11). Nevertheless, limited research has been conducted to investigate the impact of the time interval between repeated punctures and the size of thyroid nodules on the diagnostic outcome (12). Consequently, there is currently no established consensus regarding the management of thyroid nodules with inconclusive initial cytology after the initial FNA, the objective of this study is to conduct a comparative analysis between CNB and rFNA in diagnosing thyroid nodules with inconclusive initial cytological diagnosis. Additionally, the study aims to assess the sampling satisfaction rate, diagnostic efficacy, and complications associated with CNB and rFNA. Furthermore, the study seeks to investigate the potential impact of repeated puncture time and the nodule size on the diagnostic efficacy of these procedures.

## Materials and methods

### Patients

The data is drawn from 471 patients with thyroid nodules. They visited West China Hospital, Sichuan University from December 2017 to May 2022 and underwent re-puncture to confirm the diagnosis due to inconclusive cytological diagnosis of the initial FNA (in another hospital or our hospital), which were retrospectively analyzed. Thyroid ultrasonography was performed again in the above patients, and ultrasound revealed unknown or suspicious malignancy of the nodules, and CNB (n = 242) or rFNA (n = 229) was used for re-puncture.

### CNB and rFNA procedures

Both CNB and rFNA procedures were performed by sonographers with more than 10-year experience in thyroid nodule puncture. It was performed under ultrasound guidance using a Mindray Resona 7 ultrasound machine with a probe frequency of 5 to 12 MHz. Repeated puncture targets the same thyroid nodule as the initial FNA.

For rFNA cytology, mainly used were needle gauged from 21 to 25. Inserted was the needle parallel to the ultrasound probe. Thyroid samples were aspirated by moving the needle back and forth for 5 to 10 seconds, and the aspirate was immediately spread on a slide and fixed in 95% alcohol.

CNB was performed using a Bard magnum automated biopsy gun from Bard with an 18G needle. Local anesthesia was performed with 1% lidocaine on the puncture pathway, and the thyroid nodules close to the surrounding important structures (trachea, esophagus and great vessels of the neck) were injected with normal saline to form an isolation zone between the thyroid gland and the surrounding structures. Under the ultrasound guidance, the needle was punctured before the thyroid nodules, after adjusting the position and direction, the trigger was pulled to obtain tissue samples of the nodules and was operated for two to four times. The samples were placed in 10% formalin for examination, and finally the puncture area was observed for complications, and ultrasound images were stored during the puncture process.

### Pathologic analysis

The re-puncture results were completed by multiple pathologists specializing in the thyroid category in our hospital. Cytological reports were based on the TBSRTC system (13) and the CNB pathology reports were based on the criteria of the Korean Thyroid Society (11). Both rFNA cytology and CNB histology results were in 6 categories: no diagnosis or unsatisfactory (Category I), benign (Category II), atypia of unknown significance/follicular lesions of unknown significance (AUS/FLUS)(Category III), follicular neoplasm/suspicious follicular neoplasm (FN/SFN) (Category IV), suspicious malignancy (Category V), and malignant (Category VI) (11, 13). Repeated puncture results of category I were considered unsatisfactory specimens, and category II-VI were considered satisfactory specimens. Results of benign (Category II), suspicious malignant tumors (Category V), and malignant tumors (Category VI) were defined as “diagnostic results” and others as “ inconclusive results” (14, 15).

### Diagnostic performances of repeat biopsy

After undergoing postoperative pathological examination, the nodules were classified as either benign or malignant. In order to evaluate the diagnostic efficacy of CNB and rFNA in identifying malignancy, the outcome of each nodule was compared to the postoperative pathological findings. These findings were obtained from two pathologists with a minimum of five years of specialized experience in the domain of thyroid pathology.

### Statistical analysis

SPSS 22 statistical software was used. Continuous variables were presented as median (interquartile range) and was evaluated by independent t-test or Mann Whitney U test for two samples. Categorical variables were presented as frequencies and percentages and were evaluated by Chi-square test or Fischer ‘s exact test. The satisfactory rate and confirmed rate of CNB and rFNA were calculated. Compared to postoperative pathological findings, the diagnostic accuracy, sensitivity, specificity, positive predictive value (PPV) and negative predictive value (NPV) of CNB and rFNA in the diagnosis of malignant tumors were calculated, and P < 0.05 was considered statistically significant. The diagnostic accuracy of the two methods, CNB and rFNA, were compared using MedCalc v19.20 (MedCalc Software, Ostend, Belgium).

## Results

There were no statistically significant variations in terms of age or gender observed among the patients belonging to the CNB and rFNA groups. The distribution of initial FNA cytology was nondiagnostic or unsatisfactory (n = 202), and the ultrasound images suggested malignancy but the FNA results were TBSRTC category II (n = 17), Category III (n = 220), Category IV (n = 32) (**Tables 1**, **2**). After repeated puncture, the satisfactory rate of CNB sampling was higher than that of rFNA (83.9% vs 66.8%, P < 0.05).

**Table 1:** Characteristic of study population.

Characteristics	Total(n= 471)	CNB(n= 242)	rFNA(n=229)	P value
Demographics				
Age^a^, years	44(17)	43(17)	45(16)	0.069
Sex				
Female	384	197	187	0.943
male	87	45	42	
Initial FNA results				
Category I	202	98	104	0.000*
Category II	17	16	1	
Category III	220	118	102	
Category IV	32	10	22	
Maximum diameter of thyroid nodule				
<1cm	290	161	129	0.001*
1cm≤, <2cm	134	69	65	
≥2cm	47	12	35	

CNB, core needle biopsy; rFNA, repeat fine-needle aspiration.

a Expressed as Mean (interquartile range).

*P Value of <0.05 was considered statistically significant.

**Table 2:** Results and satisfaction rate of sampling after repeated puncture.

Repeat biopsy results	CNB(n= 242)	rFNA(n=229)	P value
Category I	39	76	
Category II	24	38	
Category III	24	51	
Category IV	8	20	
Category V	29	10	
Category VI	118	34	
Sampling effect			
Satisfied	203(83.9%)	153(66.8%)	0.000*
Not satisfied	39(16.1%)	76(33.2%)	
Diagnostic performances of repeat biopsy			
Inconclusive results^a^	71(29.3%)	147(64.2%)	0.000*
Diagnostic results^b^	171(70.7%)	82(35.8%)	

*P Value of <0.05 was considered statistically significant.

a Inconclusive results included categories I, III, and IV.

b Diagnostic results included categories II, V, and VI.

### Results of repeat biopsy

**Table 3** displays the distribution of repeat biopsy results based on the initial cytological categories. Diagnosis was obtained in 253 of 471 patients (53.7%). In a total of 471 patients, the rate of diagnosis was significantly higher in the CNB group than in the rFNA group (70.7% vs 35.8%, P < 0.001) (**Tables 2**). Diagnostic results obtained in the CNB and rFNA groups were compared in four categories (I, II, III, and IV) of the initial FNA cytology. For patients with category I (62.2% vs 26.9%) and category III (78.8% vs 47.1%) in the initial FNA, the CNB group had significantly higher diagnostic results than the rFNA group. Out of the total of 17 nodules displaying Ultrasound-Pathology discordant findings, which were initially diagnosed as category II, 12 were subsequently identified as malignant (category V or VI) through CNB and were further validated as malignant through postoperative pathology assessments.

**Table 3:** Results of repeat biopsy according to the initial FNA cytology.

Initial FNA		Repeat biopsy		P value
		CNB(n= 242)	rFNA(n=229)	
Category I(n= 202)	Inconclusive results^a^	37(37.8%)	76(72.1%)	0.000*
	Diagnostic results^b^	61(62.2%)	28(26.9%)	
	Category I	26	52	78
	Category II	12	21	33
	Category III	8	17	25
	Category IV	3	7	10
	Category V	13	2	15
	Category VI	36	5	41
Category II(n= 17)	Inconclusive results	4	1	0.294
	Diagnostic results	12	0	
	Category I	0	0	0
	Category II	0	0	0
	Category III	4	1	5
	Category IV	0	0	0
	Category V	4	0	4
	Category VI	8	0	8
Category III (n= 220)	Inconclusive results	25(21.2%)	54(52.9%)	0.000*
	Diagnostic results	93(78.8%)	48(47.1%)	
	Category I	12	17	28
	Category II	10	12	22
	Category III	11	29	40
	Category IV	2	8	10
	Category V	11	7	18
	Category VI	72	29	101
Category IV (n=32)	Inconclusive results	5(50.0%)	16(72.3%)	0.394
	Diagnostic results	5(50.0%)	6(27.3%)	
	Category I	1	7	8
	Category II	2	5	8
	Category III	1	4	5
	Category IV	3	5	7
	Category V	1	1	2
	Category VI	2	0	2

*P Value of <0.05 was considered statistically significant.

a Inconclusive results included categories I, III, and IV.

b Diagnostic results included categories II, IV, and VI.

**Table 4** displays the distribution of repeat biopsy outcomes based on the largest diameter of thyroid nodules. In patients with nodule maximum diameters ranging from 5 mm to less than 8 mm (72.7% vs 29.5%, P < 0.001), 8 mm to less than 10 mm (75.0% vs 44.7%, P < 0.05), and 10 mm to less than 20 mm (74.6% vs 40.0%, P < 0.001), the diagnostic accuracy was significantly higher in the CNB group compared to that in the rFNA group. There was no significant difference in the diagnostic effect between the CNB group and rFNA when the maximum diameter of the nodule was < 5 mm (55.6% vs 0%) and when the maximum diameter of the nodule was > 20 mm (41.7% vs 34.3%).

**Table 4:** Diagnostic results of repeated puncture for different sized nodules.

Maximum diameter of thyroid nodule		Repeat biopsy		P value
		CNB(n= 242)	rFNA(n=229)	
3mm≤, <5mm(n=22)	Inconclusive results^a^	8(44.4%)	4(100%)	0.096
	Diagnostic results^b^	10(55.6%)	0(0%)	
	Category I	7	1	
	Category II	2	0	
	Category III	1	2	
	Category IV	0	1	
	Category V	0	0	
	Category VI	8	0	
5mm≤, <8mm(n=177)	Inconclusive results	27(27.3%)	55(70.5%)	0.000*
	Diagnostic results	72(72.7%)	23(29.5%)	
	Category I	19	29	
	Category II	8	9	
	Category III	8	18	
	Category IV	0	8	
	Category V	14	4	
	Category VI	50	10	
8mm≤, <10mm(n=91)	Inconclusive results	11(25.0%)	26(55.3%)	0.03
	Diagnostic results	33(75.0%)	21(44.7%)	
	Category I	7	17	
	Category II	5	7	
	Category III	4	8	
	Category IV	0	1	
	Category V	4	1	
	Category VI	24	13	
10mm≤, <20mm(n=134)	Inconclusive results	18(25.4%)	39(60.0%)	0.000*
	Diagnostic results	53(74.6%)	26(40.0%)	
	Category I	4	19	23
	Category II	6	12	19
	Category III	10	17	27
	Category IV	4	3	6
	Category V	11	5	16
	Category VI	2	2	4
≥20mm(n=47)	Inconclusive results	7(58.3%)	23(65.7%)	0.912
	Diagnostic results	5(41.7%)	12(34.3%)	
	Category I	2	10	12
	Category II	3	10	13
	Category III	1	6	7
	Category IV	4	7	11
	Category V	0	0	0
	Category VI	2	2	4

*P Value of <0.05 was considered statistically significant.

a Inconclusive results included categories I, III, and IV.

b Diagnostic results included categories II, IV, and VI.

**Table 5** presents the distribution of repeat biopsy results based on the repeat biopsy interval. The diagnostic rate was higher in the CNB group than in the rFNA group at intervals of < 90 days (79.8% vs 26.1%, P < 0.001), 90 – 180 days (57.1% vs 36.1%, P < 0.05), and 180 days – one year (66.7% vs 38.5% P < 0.001). At intervals > one year (69.6% vs 58.3%, P > 0.05), there was no significant difference in the diagnostic effect between CNB and rFNA.

**Table 5:** Diagnostic results of repeated puncture at different intervals.

Repeated Puncture Interval		Repeat biopsy		P value
		CNB(n= 242)	rFNA(n=229)	
< 90 days(n=145)	Inconclusive results^a^	20(20.2%)	34(73.9%)	0.000*
	Diagnostic results^b^	79(79.8%)	12(26.1%)	
	Category I	7	22	
	Category II	8	4	
	Category III	8	6	
	Category IV	5	6	
	Category V	16	2	
	Category VI	55	6	
90-180 days(n=168)	Inconclusive results	21(42.9%)	76(63.9%)	0.012*
	Diagnostic results	28(57.1%)	43(36.1%)	
	Category I	16	36	
	Category II	3	21	
	Category III	4	33	
	Category IV	1	7	
	Category V	5	5	
	Category VI	20	17	
180 day-1year(n=100)	Inconclusive results	16(33.3%)	32(61.5%)	0.005*
	Diagnostic results	32(66.7%)	20(38.5%)	
	Category I	10	16	
	Category II	6	10	
	Category III	6	11	
	Category IV	0	5	
	Category V	3	2	
	Category VI	24	13	
> 1 year (n=58)	Inconclusive results	14(30.4%)	5(41.7%)	0.694
	Diagnostic results	32(69.6%)	7(58.3%)	
	Category I	6	2	
	Category II	7	3	
	Category III	6	1	
	Category IV	2	2	
	Category V	5	1	
	Category VI	20	3	

*P Value of <0.05 was considered statistically significant.

a Inconclusive results included categories I, III, and IV.

b Diagnostic results included categories II, IV, and VI.

### Comparing the repeat biopsy results to postoperative pathological findings

The diagnostic performance of rFNA versus CNB for malignant thyroid nodules was evaluated by analyzing the results of repeat biopsy results alongside postoperative pathological findings (**Tables 6**, **7**). In the CNB and rFNA groups, respectively, 172 and 88 patients underwent surgery. **Table 6** shows the postoperative pathology for each category of repeat puncture findings (categories I - VI). There was a higher diagnostic accuracy in the CNB group than in the rFNA group, 89.3% and 79.5%, respectively (area under the curve =0.945 and 0.814, respectively, p<0.05). The incidence of inconclusive diagnosis (repeat puncture results in categories I, III, and IV) was significantly lower in the CNB group than in the rFNA group(9.88% vs 35.22%, p<0.05). CNB and rFNA diagnosis of category V or VI nodules confirmed by postoperative pathology as malignant.

**Table 6:** Repeat puncture results and postoperative pathological findings.

Repeat biopsy results	Postoperative pathological findings	Repeat biopsy methods	
		CNB (n=172)	rFNA (n=88)
Category I	Benign	1	4
	Malignancy	6	6
Category II	Benign	4	11
	Malignancy	4	2
Category III	Benign	1	2
	Malignancy	4	16
Category IV	Benign	1	1
	Malignancy	4	2
Category V	Benign	0	0
	Malignancy	29	10
Category VI	Benign	0	0
	Malignancy	118	34

**Table 7:** Diagnostic performances of repeat biopsy.

Outcomes	CNB (n=172)		rFNA (n=88)	
	Malignant	Non-malignant	Malignant	Non-malignant
Category V–VI	147	0	44	0
Category I–IV	18	7	26	18
Diagnostic performance	Incidence		Incidence	
Diagnostic accuracy	89.3%		70.5%	
Area under the curve	0.945		0.814	
Inconclusive diagnosis^a^	9.88%		35.22%	
Sensitivity	89.1%		62.9%	
Specificity	100%		100%	
Positive predictive value	100%		100%	
Negative predictive value	28.0%		40.1%	

a Inconclusive results included categories I, III, and IV.

### Complications

There were 68 minor bleeds after CNB and 7 minor bleeds after rFNA, and all bleeding cases were absorbed after 30 minutes of body surface compression without other clinical interventions. No case reported serious complications such as infection, severe hematoma, nerve injury, or tumor seeding. Nodules immediately adjacent to the capsule had a bleeding probability of 22.7% after receiving CNB and nodules not adjacent to the capsule had a bleeding probability of 37.0%. Not immediately adjacent to the capsule was a risk factor for nodular puncture bleeding (P < 0.05).Adler flow grade of CNB nodules was not a risk factor for bleeding (P > 0.05). Nodular bleeding after CNB puncture is shown in **Table 8**.

**Table 8:** Nodal bleeding after CNB puncture.

Characteristics	No bleeding(n=174)	Hemorrhage(n=68)	P value
Blood flow grade			0.788
Alder 0	59	21	
Alder 1	93	40	
Alder 2	18	5	
Alder 3	4	2	
Relation to capsule			0.016*
Un-adjacent capsule	58(33.3%)	34(50.0%)	
Immediately adjacent to capsule	116(66.7%)	34(50.0%)	

*P Value of <0.05 was considered statistically significant.

## Discussion

Thyroid nodules are prevalent clinical conditions, and ultrasound serves as the preferred modality for imaging thyroid diseases. FNA offers the advantages of safety and cost-effectiveness, enabling the identification of lesion characteristics to a certain extent, thereby preventing unnecessary diagnostic surgeries and facilitating informed treatment decisions for patients (16, 17). Nonetheless, this technique is not without limitations, as its outcomes heavily depend on the operator’s proficiency in performing the puncture and the expertise of the pathologist in interpreting the results. FNA is limited by its reliance on sampled specimens, which only allows for cytomorphology examination, lacking the ability to assess histological characteristics and additional information.In approximately 20% of thyroid nodule cases, rFNA procedures may yield non-diagnostic or indeterminate results, thereby contributing to a relatively substantial proportion of indeterminate results (18). The diagnostic accuracy for thyroid follicular carcinoma and rare malignancies such as lymphoma, anaplastic carcinoma, medullary carcinoma, and metastasis is relatively low (19–23).

As a result, there has been a clinical proposal to adopt CNB as an alternative to FNA. Following repeated punctures, the overall satisfaction with sampling was found to be higher in CNB compared to rFNA. This trend was consistent across all subgroups within categories I, III, and IV. In the current investigation, it was found that 53.7% of the repeated punctures yielded a conclusive diagnosis. Moreover, the CNB group exhibited a notably lower rate of inconclusive diagnoses compared to the rFNA group, particularly in cases where the initial FNA yielded diagnoses falling within categories I and III. This result was similar to that reported in Seon et al. (15) study.

The correlation between nodule size and nondiagnostic or indeterminate outcomes is well-established in the literature (24–26), and currently, nodule size is utilized to determine the need for FNA or CNB. However, while current guidelines for thyroid nodule management do not routinely recommend puncture for nodules smaller than 10 mm (1, 17, 27–29), this approach may result in delayed diagnosis for some patients with nodules smaller than10mm but that exhibit more aggressive characteristics. Hyeon et al. (30) discovered a substantial false negative rate of FNA in large nodules. Additionally, it is possible that the true false negative rate of small nodules is underestimated due to the fact that patients with small nodules and benign results typically do not undergo thyroidectomy (30). In a study conducted by Jung Hyun Yoon et al. (26) regarding FNA and CNB, it was observed that the diagnostic sensitivity of CNB surpassed that of FNA specifically within the subgroup of lesions measuring less than 10 mm. In our study, CNB was conducted on thyroid nodules measuring less than 10 mm, as a result of identifiable malignancy indicators observed on ultrasound images, which have the potential to contribute to an unfavorable prognosis. These indicators included the presence of multiple suspicious nodules, suspicious nodules situated in the isthmus of the thyroid gland, invasion of the pericardium of the thyroid gland, or neck lymph node abnormalities. Therefore, careful consideration must be given to the use of CNB for nodules smaller than 10 mm in order to obtain a more definitive diagnosis.

This study compared the results of repeated puncture in thyroid nodules of varying sizes. When considering nodules of insufficient size (<5 mm) or excessive size (> 20 mm), the diagnostic efficacy between the CNB group and the rFNA group exhibited minimal disparity. However, the CNB group exhibited a significantly lower rate of indeterminate diagnosis compared to the rFNA group in patients presenting with nodules ranging from 5 mm to 20 mm in maximum diameter. These findings suggest that CNB may be the preferred method for re-puncture of small thyroid nodules with a maximum diameter of 5-10 mm. Furthermore, this finding demonstrates that CNB remains effective in diagnosing nodules smaller than 10 mm, aligning with the perspective of Jung et al. (26) CNB is not suitable in all cases and does not provide a higher diagnostic yield for nodules that are too small, in addition to other risks. Therefore, more rigorous selection of patients for CNB is needed. When dealing with small nodules, the puncture operator should exercise caution regarding the surrounding structures of the thyroid gland, effectively mitigating risks and enhancing the safety of the procedure. For instance, the implementation of normal saline injection around the thyroid gland to establish an isolation zone can be employed.

Furthermore, the present study revealed that among patients undergoing repeat puncture procedures with intervals of less than 1 year, the incidence of inconclusive diagnostic outcomes was significantly lower in the CNB group compared to the rFNA group. In a study conducted by Rosemary A et al. (31) examines the histological alterations observed in needle tract formation subsequent to FNA puncture of thyroid nodules. The identified changes encompass hemorrhage, granulation, exuberant fibroblast reaction, reactive follicular cells, infarction, and scar formation. It is noteworthy that the presence of reactive follicular cells displaying nuclear grooving and nuclear clearance, which are atypical cellular changes resulting from prior FNA surgeries, bear resemblance to the cytological characteristics observed in the pathology of papillary thyroid carcinoma. Correspondingly, Chetna Sharma et al. (32) discovered modifications pertaining to nuclear clearance, nuclear anisotropy, and other factors that may challenge traditional diagnostic methods. The aforementioned alterations have been observed to reach their maximum occurrence within a period of 20-40 days following FNA surgery. Clinicians and pathologists should maintain awareness of the diagnostic challenges arising from atypical cellular alterations linked to prior FNA procedures, despite their infrequent occurrence.

Although the diagnosis of thyroid nodules can typically be achieved through morphological examination using hematoxylin and eosin staining, the utilization of molecular markers has been recognized as a valuable tool in distinguishing between benign and malignant nodules in cases where differentiation is challenging. In a comprehensive study by Seulki Song et al. (33), detection of Galectin-3, Hector Battifora mesothelial cell-1, and cytokeratin-19 markers in post-puncture samples significantly improved the diagnosis of indistinguishable nodules. Erik Kouba’s (34) research showed that next-generation sequencing techniques can identify BRAF V600E mutations, thereby facilitating the characterization of tumors that present histologic alterations that hinder the attainment of a conclusive tissue diagnosis. In this study, the pathologist made a diagnosis of malignancy when a significant quantity of diverse follicular pithelial cells were observed under microscopic examination following repeated puncture, or when a limited quantity of diverse follicular epithelial cells were accompanied by positive molecular markers, immunohistochemistry, and the presence of BRAF V600E and Telomerase reverse transcriptase (TERT) genes mutation.

Our study primarily examined the TBSRTC category of pathological diagnoses following repeat puncture, where atypical changes after the initial puncture may be predominantly in category III and category IV. The classification of malignant diagnoses encompasses Category V and Category VI. The present study observed that the postoperative pathology of all identified nodules with malignancy was consistent with papillary thyroid carcinoma. Therefore, upon conducting a comparative analysis of the diagnostic confirmation rate following repeated punctures using both methods, it was observed that chronic pathological alterations occurring within intervals of less than 90 days had minimal impact on the confirmation rate, specifically within categories II,V and VI.These approaches aims to partially alleviate the consequences of the initial puncture. For patients with a strong desire for the diagnosis of thyroid nodules, but have inconclusive initial FNA results, CNB re-puncture at intervals < 90 days may be considered clinically. However, the diagnostic accuracy is significantly higher for categories V and VI, whereas for categories III and IV, the potential influence of chronic pathological alterations subsequent to the initial puncture cannot be entirely disregarded. Further comprehensive investigations are warranted to explore the pathological changes instigated within the first 90 days following the puncture.

In order to evaluate the effectiveness of repeat biopsy in detecting malignancy, this study has compared the results of repeat biopsy with the postoperative pathological findings. The CNB group exhibited a higher level of accuracy in diagnosing malignancy compared to the rFNA group. Additionally, the CNB group demonstrated a significantly lower incidence of uncertain diagnoses (categories I, III, and IV) in comparison to the rFNA group. Hence, the aforementioned findings indicate that CNB can significantly contribute to the reduction of non-diagnostic and equivocal diagnoses resulting from FNA, thereby preventing unnecessary diagnostic procedures.

Following CNB, a total of 68 patients (28.1%) experienced minor bleeding, while seven patients experienced bleeding after rFNA. However, all instances of bleeding were resolved through 30 minutes of body surface compression without requiring additional clinical interventions. Notably, no instance of serious complications, such as infection, severe hematoma, nerve injury, or tumor seeding, was reported. The increased incidence of minor bleeding observed in this study may be attributed to the predominance of punctured nodules with a maximum diameter of less than 10 mm, coupled with the abundant blood flow within the thyroid tissue. Our research found that the absence of immediate adjacency to the capsule was identified as a significant risk factor for hemorrhage in nodules with pre-existing hemorrhage. The potential cause of this phenomenon could be attributed to the heightened probability of the needle tip traversing a greater amount of the thyroid tissue and coming into contact with blood vessels within the gland while puncturing nodules that are not in direct proximity to the capsule. It is worth noting that the Adler flow grade of CNB nodules did not demonstrate a significant association with the occurrence of bleeding. To gain a deeper understanding of post-CNB hemorrhage, future studies should consider potential risk factors like thyroid nodule hardness more comprehensively. It’s crucial to expand the sample size to get more statistically significant results for accurate assessment of associated factors.

Common minor complications associated with CNB encompass mild to moderate hematoma, bleeding, carotid artery injury, temporary voice alterations, puncture-related tracheal incidents, and dysphagia (35–37). Complications associated with CNB have been documented to manifest in a range of 0.2% to 1.0% (17, 35, 38, 39). Furthermore, there exists no substantial disparity in terms of patient discomfort and tolerability between FNA and CNB (39, 40). Prior to performing the CNB puncture, local anesthesia was administered using 1% lidocaine over the puncture access. Certain patients may experience mild or radiating pain, which is generally well-tolerated and gradually subsides following the puncture procedure. These findings collectively affirm the safety and acceptability of CNB as a medical procedure. Furthermore, it is noteworthy that in our hospital, the cost of CNB is 16% higher compared to FNA. Therefore, when dealing with patients whose initial puncture results in an inconclusive diagnosis, it is imperative to provide patients with comprehensive information regarding the disparities in sampling techniques between CNB and FNA, as well as the associated price discrepancy, prior to considering a repeat puncture. It is important to acknowledge that while CNB allows for a larger sample size, it also incurs higher expenses and greater risk of haemorrhage.

In contrast to FNA, CNB offers distinct advantages, such as the ability to promptly evaluate the adequacy of the obtained specimen during sampling, thereby allowing for immediate re-puncture in cases of inadequacy. Using CNB to collect a substantial quantity of tissue from the lesion allows for the acquisition of more histological information, including the nodular capsule, the correlation between the nodule and the surrounding thyroid tissue, and immunohistochemical data (10, 41). Moreover, CNB is less dependent on the skill of the puncture physician if the needle is successfully pierced into the thyroid nodule.

Although diagnostic surgery can definitively diagnose the nature of thyroid nodules, it may increase the complication rate and mortality in the elderly patients and may delay the management of patients with anaplastic thyroid carcinoma or lymphoma (21, 42, 43). Therefore, accurate preoperative diagnosis of thyroid nodules is crucial for effective clinical decision-making.

There are several limitations to this study. This study is a retrospective, non-randomized study and inevitably suffers from selection bias that may affect the study results, especially the operators of the initial FNA being different physicians in different hospitals. Moreover, assessing the precise diagnostic accuracy of malignant tumors is an intricate task that requires careful examination of histomorphological changes following pre-existing FNA, which is an integral part of our ongoing investigation and needs further refinement. The behavior of most papillary thyroid microcarcinomas is indolent, exhibiting minimal changes over extended periods of follow-up. Consequently, some patients may be initially misdiagnosed as benign after repeated biopsies and subsequently not receive regular monitoring, potentially leading to an underestimation of certain malignant nodules. Our intention is to conduct further studies in order to observe these patients for an extended period of time. This study also requires further research on the complications associated with CNB, especially bleeding. We need to expand the study to include more potential factors to further validate our findings and provide more comprehensive data support. Furthermore, we will proceed to develop pain assessment tools, such as pain scales, to comprehensively evaluate the patients’ subjective experience of pain following CNB and FNA puncture.

In conclusion, the ultrasound-guided CNB sampling demonstrated a higher satisfactory rate and diagnostic rate of specimens compared to rFNA. Furthermore, our findings indicate that the diagnostic efficacy of CNB puncture remains unaffected by the time interval and the nodule size. It is important to note that CNB is a safe procedure, and the risk of bleeding from CNB puncture nodules is associated with their proximity to the capsule. Consequently, CNB serves as a valuable alternative for the management of thyroid nodules with inconclusive cytological diagnosis of initial FNA during re-puncture.

## Data availability statement

The original contributions presented in the study are included in the article/supplementary material. Further inquiries can be directed to the corresponding author.

## Ethics statement

The studies involving humans were approved by Ethics Committee of West China Hospital, Sichuan University. The studies were conducted in accordance with the local legislation and institutional requirements. The human samples used in this study were acquired from a by- product of routine care or industry. Written informed consent for participation was not required from the participants or the participants’ legal guardians/next of kin in accordance with the national legislation and institutional requirements.

## Author contributions

XS: Conceptualization, Data curation, Formal analysis, Writing – original draft. CY: Conceptualization, Methodology, Writing – original draft. WY: Data curation, Methodology, Writing – original draft. BM: Methodology, Project administration, Supervision, Writing – review & editing.
